# Enhancing interoperability features in the Global Alzheimer's Association Interactive Network

**DOI:** 10.1002/alz70860_101704

**Published:** 2025-12-23

**Authors:** Cally Xiao, Gazzali Jaleel, Ioannis Pappas, Arthur W. Toga

**Affiliations:** ^1^ Laboratory of Neuro Imaging, Stevens Neuroimaging and Informatics Institute, Keck School of Medicine, University of Southern California, Los Angeles, CA, USA

## Abstract

**Background:**

The Global Alzheimer's Association Interactive Network (GAAIN) was established in 2013 as the first federated Alzheimer's disease data sharing platform of its kind. To enhance the interoperability of the GAAIN Interrogator, which allows users to explore data collected across multiple sites and modalities and run preliminary regression analyses, we have established connections to external data sharing platforms to validate access and save cohort attributes.

**Method:**

Using HTTPS and authorization tokens, we set up a secure connection to the Imaging and Data Archive (IDA) hosted by the Laboratory of Neuro Imaging (LONI). This connection allows for data exchange between the two platforms so that users logged into the GAAIN Interrogator can directly identify which Data Partners they already have approved access to via the IDA.

**Result:**

By connecting the GAAIN Interrogator to the IDA, users who have approved access to a Data Partner via the IDA can directly access the study download page without having to log in to the IDA. In addition, the subject IDs of custom cohorts defined in the Interrogator can be sent to the IDA so that users can download neuroimaging and comma separated values (CSV) data corresponding to their cohorts of interest. This process allows for a seamless transition from data discovery in the Interrogator to data access and download in the IDA.

**Conclusion:**

The development of the GAAIN‐IDA connection improves the interoperability features of the GAAIN Interrogator, which can be expanded to other external platforms such as the AD Workbench hosted by the Alzheimer's Disease Data Initiative (ADDI). The analytical capabilities and remote processing pipelines in the Interrogator can also be improved upon by building on this model, ensuring GAAIN as a key resource in the Alzheimer's disease and related dementias research community.

